# Quantitative comparison of the mean–return-time phase and the stochastic asymptotic phase for noisy oscillators

**DOI:** 10.1007/s00422-022-00929-6

**Published:** 2022-03-23

**Authors:** Alberto Pérez-Cervera, Benjamin Lindner, Peter J. Thomas

**Affiliations:** 1 National Research University Higher School of Economics, Moscow, Russia; 2 Instituto de Matemática Interdisciplinar, Universidad Complutense de Madrid, Madrid, Spain; 3 Bernstein Center for Computational Neuroscience Berlin, Institute of Physics, Humboldt University, Berlin, Germany; 4 Department of Mathematics, Applied Mathematics and Statistics, Case Western Reserve University, Cleveland, OH USA

**Keywords:** Stochastic oscillator, Phase reduction, Mean–return-time sections, Isochrons, Isostables, Neuroscience

## Abstract

Seminal work by A. Winfree and J. Guckenheimer showed that a deterministic phase variable can be defined either in terms of Poincaré sections or in terms of the asymptotic (long-time) behaviour of trajectories approaching a stable limit cycle. However, this equivalence between the deterministic notions of phase is broken in the presence of noise. Different notions of phase reduction for a stochastic oscillator can be defined either in terms of mean–return-time sections or as the argument of the slowest decaying complex eigenfunction of the Kolmogorov backwards operator. Although both notions of phase enjoy a solid theoretical foundation, their relationship remains unexplored. Here, we quantitatively compare both notions of stochastic phase. We derive an expression relating both notions of phase and use it to discuss differences (and similarities) between both definitions of stochastic phase for (i) a spiral sink motivated by stochastic models for electroencephalograms, (ii) noisy limit-cycle systems-neuroscience models, and (iii) a stochastic heteroclinic oscillator inspired by a simple motor-control system.

## Introduction

An important simplification in the analysis of nonlinear oscillators is the reduction in their dimensionality by means of a phase description. The study of oscillations by way of a phase variable facilitates the study of relevant features of an oscillator such as possible synchronization regimes, coherence, or sensitivity to perturbations (Winfree 2001; Kuramoto 2003; Pikovsky et al. 2003). In deterministic systems, oscillations often correspond to attracting limit cycles in the phase space. In these systems, the usage of the phase variable is not restricted to the limit cycle but also to the whole basin of attraction by means of the isochrons. Isochrons can be understood as Poincaré sections having the same return time (the period *T* of the oscillator itself) or as the set of points having the same asymptotic convergence to the cycle (Hirsch and Pugh 1970; Winfree 1974; Guckenheimer 1975).

Due to the increasing importance of stochastic oscillations in many biological systems, over the last decades several authors have focused on describing stochastic oscillators by means of a phase variable, applying deterministic phase concepts to stochastic systems (Freund et al. 2000, 2003; Yoshimura and Arai 2008; Teramae et al. 2009; Zhou et al. 2013; Ma et al. 2014; Bonnin 2017; Bressloff and MacLaurin 2018; Giacomin et al. 2018; Aminzare et al. 2019; Engel and Kuehn 2021; Cheng and Qian 2021). However, there are cases where noise strongly alters the dynamics of the system or oscillations may even emerge only due to noise, as for instance in excitable systems (Lindner et al. 2004) or for noisy heteroclinic oscillators (Giner-Baldo et al. 2017). In general, the stochastic case, if it is not a weak perturbation of a deterministic limit-cycle system, requires new phase concepts. Following this insight, the above-mentioned notions of deterministic phase, which are based on Poincaré sections and on the system’s asymptotic behaviour, have been generalised to stochastic systems. Whereas Schwabedal and Pikovsky (2013) proposed a notion of phase based on Poincaré sections having a uniform mean–return-time (MRT) property, Thomas and Lindner (2014) introduced an asymptotic phase for stochastic oscillators by means of the argument of the slowest decaying complex eigenfunction of the backward Kolmogorov operator (equivalently, the generator of the Markov process, or the stochastic Koopman operator). However, and in marked contrast to the deterministic case, these two notions of stochastic phase are not equivalent (Cao 2017).

In this paper, we explore the nature of the differences between these two notions of stochastic phase. To this end, we will make use of a recently discovered link between the MRT phase and the Kolmogorov backwards operator (Cao et al. 2020). By exploiting this link, we can calculate both phases using one computational framework; we will use this framework to compare systematically the differences between the MRT phase and the asymptotic phase for a number of stochastic biological systems.

Our paper is organised as follows. In Sect. 2, we review the deterministic phase and isochrons. Next, in Sect. 3 we review both notions of stochastic phase, the MRT and the asymptotic phase. Then, in Sect. 4 we show a procedure relating both expressions which we illustrate by different examples in Sect. 5. We conclude the paper with a discussion of our results. In the “Appendix”, we provide details about the numerical methods used in this paper.

## The deterministic phase

Consider an autonomous system of ODEs

1 $$\begin{aligned} \dot{\mathbf {x}} = \mathbf {F}(\mathbf {x}), \qquad \mathbf {x}\in \mathbb {R}^{n}, \qquad n \ge 2 , \end{aligned}$$

whose flow is denoted by $\phi _t(\mathbf {x})$. Moreover, we assume $\mathbf {F}(\mathbf {x})$ is a $\mathcal {C}^2$ vector field having a *T*-periodic, asymptotically stable, normally hyperbolic limit cycle parameterized by the phase variable $\theta = 2\pi t/T$

2 $$\begin{aligned} \begin{aligned} \gamma :\mathbb {T}:= \mathbb {R}/\mathbb {Z}&\rightarrow \mathbb {R}^{n}\\ \theta&\mapsto \gamma (\theta ), \end{aligned} \end{aligned}$$

Hence, the dynamics of Eq. (1) in $\varGamma$ can be reduced to a single variable system

3 $$\begin{aligned} \dot{\theta } = \frac{2\pi }{T}, \end{aligned}$$

As we study attracting limit cycles, any point $\mathbf {x}\in \mathcal {M}$, where $\mathcal {M}$ is the basin of attraction of the limit cycle $\varGamma$, will approach $\varGamma$ as $t \rightarrow \infty$ (Hirsch and Pugh 1970). Thus, two points *p* and $q \in \mathcal {M}$ will have the same asymptotic phase if

4 $$\begin{aligned} \lim _{t \rightarrow \infty } |\phi _t(q) - \phi _t(p)| = 0. \end{aligned}$$

This condition extends the notion of phase of oscillation to the basin of attraction $\mathcal {M}$ of $\varGamma$. Indeed, we can define the function

5 $$\begin{aligned} \begin{aligned} \vartheta :\mathcal {M} \subset \mathbb {R}^{n}&\rightarrow \mathbb {T}=[0,2 \pi ),\\ \mathbf {x}&\mapsto \vartheta (\mathbf {x}) = \theta . \end{aligned} \end{aligned}$$

assigning a phase to each point $\mathbf {x}\in \mathcal {M}$. We can thus define the isochrons as the level sets of $\vartheta (\mathbf {x})$, that is

6 $$\begin{aligned} \mathcal {I}_\theta = \{ \mathbf {x}\in \mathcal {M} \quad | \quad \vartheta (\mathbf {x}) = \theta \} \end{aligned}$$

which correspond to the leaves of the (strong) stable foliation (that is, the stable manifold $\mathcal {M}$) of $\varGamma$ (Winfree 1974; Guckenheimer 1975; Winfree 1980). For a normally hyperbolic invariant manifold as $\mathcal {M}$, the phaseless sets correspond to $\mathbb {R}^n {\setminus } \mathcal {M}$.

## Stochastic phase notions

Next, we review two notions of phase for stochastic systems: the stochastic asymptotic phase and the mean–return-time (MRT) phase. Throughout this Section, we will consider Langevin systems

7 $$\begin{aligned} \frac{\mathrm{d}\mathbf {X}}{\mathrm{d}t}=\mathbf {f}(\mathbf {X}) + \mathbf {g}(\mathbf {X})\xi (t) \end{aligned}$$

where $\mathbf {f}$ is an *n*-dimensional $C^2$ vector field, $\mathbf {g}$ is a $C^2$ $n\times k$ matrix, and $\xi$ is *k*-dimensional white noise with uncorrelated components $\langle \xi _i(t)\xi _j(t')\rangle =\delta (t-t')\delta _{i,j}$. Moreover, we require the elements $g_{ij}(\mathbf {x})$ in $\mathbf {g}$ to be such that the matrix $\mathcal {G}=\frac{1}{2} gg^\top$ is invertible for all $\mathbf {x}\in \mathbb {R}^n$ (see Sect. 4 for further details). For mathematical convenience, we interpret the stochastic differential equation Eq. (7) in the sense of Itô (Gardiner 1985).

### The mean–return-time stochastic phase

Schwabedal and Pikovsky (2013) introduced a definition for the phase of a stochastic oscillator in terms of a system of Poincaré sections $\{ \ell _{\mathrm{MRT}}(\phi ), 0 \le \phi \le 2\pi \}$, foliating a domain $\mathcal {R} \subset \mathbb {R}^2$ and possessing a MRT property: a section $\ell _{\mathrm{MRT}}$ satisfies the MRT property if for all the points $\mathbf {x}\in \ell _{\mathrm{MRT}}$ the mean return time from $\mathbf {x}$ back to $\ell _{\mathrm{MRT}}$, having completed one full rotation, is constant.

First defined by Schwabedal and Pikovsky (2013) by means of an algorithmic numerical procedure, the MRT phase was recently related to the solution of a boundary value problem (Cao et al. 2020). As the authors in this paper showed, the $\ell _{\mathrm{MRT}}$ sections correspond to the level curves of a function $T(\mathbf {x})$, with appropriate boundary conditions, satisfying the following PDE associated with a first-passage-time problem

8 $$\begin{aligned} \mathcal {L}^\dagger T(\mathbf {x}) = -1, \end{aligned}$$

where $\mathcal {L}^\dagger$ corresponds to the Kolmogorov backwards operator (the adjoint of the Kolmogorov forward operator $\mathcal {L}$). Both operators read

9 $$\begin{aligned} \mathcal {L}[u(\mathbf {x})]&= - \nabla \cdot (\mathbf {f}(\mathbf {x}) u(\mathbf {x})) + \sum _{i,j} \partial _i \partial _j(\mathcal {G}_{ij}(\mathbf {x}) u(\mathbf {x})) \end{aligned}$$

10 $$\begin{aligned} \mathcal {L}^\dagger [u(\mathbf {x})]&= \mathbf {f}(\mathbf {x})\cdot \nabla u(\mathbf {x}) + \sum _{i,j} \mathcal {G}_{ij}(\mathbf {x})\partial _i \partial _j u(\mathbf {x}), \end{aligned}$$

where $\mathcal {G}=\frac{1}{2} gg^\top$, and *u* is an arbitrary $C^2$ function.

Cao et al. (2020) showed that upon imposing a boundary condition amounting to a jump by $\overline{T}$ (the mean period of the oscillator) across an arbitrary section transverse to the oscillation, the *unique* solution of Eq. (8), up to an additive constant $T_0$, is a version of the so-called MRT function,

11 $$\begin{aligned} \varTheta (\mathbf {x})=(2\pi / \overline{T})(T_0-T(\mathbf {x})). \end{aligned}$$

Hence, the MRT phase $\varTheta (\mathbf {x})$ satisfies

12 $$\begin{aligned} \mathcal {L}^\dagger \varTheta (\mathbf {x}) = \frac{2 \pi }{\overline{T}}, \end{aligned}$$

so it evolves in the mean as

13 $$\begin{aligned} \frac{\mathrm{d}}{\mathrm{d}t} \mathbb {E}[\varTheta (\mathbf {x})] = \frac{2 \pi }{\overline{T}}, \end{aligned}$$

which is formally analogous to the dynamics for the deterministic phase (see Eq. 3).

### The stochastic asymptotic phase

Thomas and Lindner (2014) defined a notion of stochastic asymptotic phase by means of the eigenfunctions of the Kolmogorov backwards operator. Since the Kolmogorov backwards operator and the stochastic Koopman operator are equivalent (Črnjarić-Žic et al. 2019), the setup in Thomas and Lindner (2014), which we next review, generalises the Koopman approach to obtain the phase of deterministic oscillators to stochastic systems (Mauroy and Mezić 2018; Kato et al. 2021).

Consider an ensemble of trajectories described by means of the conditional density

$$\begin{aligned} \rho (\mathbf {y},t\,|\,\mathbf {x},s)=\frac{1}{|\mathrm{d}\mathbf {y}|}\Pr \left\{ \mathbf {X}(t)\in [\mathbf {y},\mathbf {y}+\mathrm{d}\mathbf {y})\,|\,\mathbf {X}(s)=\mathbf {x}\right\} \end{aligned}$$

for $s<t$. The density evolves following Kolmogorov’s equations

14 $$\begin{aligned} \underline{} \frac{\partial }{\partial t}\rho (\mathbf {y},t\,|\,\mathbf {x},s)=\mathcal {L}_\mathbf {y}[\rho ], -\frac{\partial }{\partial s}\rho (\mathbf {y},t\,|\,\mathbf {x},s)=\mathcal {L}^\dagger _\mathbf {x}[\rho ] \end{aligned}$$

for $\mathcal {L}$ and $\mathcal {L}^\dagger$ defined in Eq. (9). Assuming the operators $\mathcal {L}$, $\mathcal {L}^\dagger$ admit a complete biorthogonal eigenfunction expansion with respect to the standard inner product $\langle u\,|\,v\rangle =\int _{\mathbb {R}^n}u^*(\mathbf {x})v(\mathbf {x})\,d\mathbf {x}$ (where $u\in C^2\cap L_\infty$ and $v\in C^2\cap L_1$)

15 $$\begin{aligned} \mathcal {L}[P_\lambda ]=\lambda P_\lambda ,\quad \mathcal {L}^\dagger [Q^*_\lambda ]=\lambda Q^*_\lambda ,\quad \langle Q_\lambda \,|\,P_{\lambda '}\rangle =\delta _{\lambda \lambda '} \end{aligned}$$

we can write the conditional density as a sum

16 $$\begin{aligned} \rho (\mathbf{y} ,t|\mathbf{x} ,s) = P_0(\mathbf{y} ) + \sum _{\lambda \not =0} e^{\lambda (t-s)} P_\lambda (\mathbf{y} ) Q^*_\lambda (\mathbf{x} ), \end{aligned}$$

with $P_0$ suitably normalized, representing the unique stationary probability distribution. The normalization condition in Eq. (16) implies $Q_0\equiv 1$.

The construction of the stochastic asymptotic phase requires one to assume several properties of the system (7) which Thomas and Lindner termed “robustly oscillatory”. First, we require that the nontrivial eigenvalue in Eq. (15) with least negative real part $\lambda _1 = \mu + i\omega$ is complex, and unique (occurs with algebraic multiplicity one). Thus, we can express its associated right (forward) and left (backward or adjoint) eigenfunctions in polar form as $P_{\lambda _1}(\mathbf{y} ) = v(\mathbf {y})e^{-i\phi (\mathbf {y})}$ and $Q^*_{\lambda _1}(\mathbf{x} ) = u(\mathbf {x})e^{i\psi (\mathbf {x})}$, where $v(\mathbf {y})\ge 0$ and $u(\mathbf {x})\ge 0$ are real functions specifying the amplitude of the corresponding eigenfunction.

Second, we require that all other nontrivial eigenvalues $\lambda '$ be significantly more negative, that is, $\Re [\lambda '] < 2\mu$. This condition guarantees that at sufficiently long times, the sum in Eq. (16) may be written as

$$\begin{aligned} \frac{\rho (\mathbf{y} ,t|\mathbf{x} ,s) - P_0(\mathbf{y} )}{2 v(\mathbf {y})u(\mathbf {x})} \approx e^{\mu (t-s)}\cos (\omega (t-s) + \psi (\mathbf {x}) - \phi (\mathbf {y})). \end{aligned}$$

This asymptotic form means that the density approaches its steady state as a damped focus, with an oscillation period of $2\pi /\omega$, and a decaying amplitude with time constant $1/|\mu |$.

The third assumption is heuristic rather than rigorous: the description as a “stochastic oscillator” will be more appropriate, the larger the quality factor $|\omega /\mu |$ (Giner-Baldo et al. 2017). That is, provided the oscillation completes sufficiently many rotations before the damping reduces its phase coherence beyond detectability, the system will be “robustly oscillatory”. Thus, we require $|\omega /\mu | \gg 1$, without specifying an explicit threshold for this quantity.

The dynamics of this focus, capturing the *asymptotic* oscillatory behaviour of the system, can be obtained in an alternative way: along trajectories $\mathbf {X}(t)$, the slowest decaying modes $Q^*_{\lambda _1}(\mathbf {X}(t))$ evolve in the mean as

17 $$\begin{aligned} \frac{\mathrm{d}}{\mathrm{d}t}\mathbb {E}[Q^*_{\lambda _1}] = \lambda _1\mathbb {E}[Q_{\lambda _1}^*], \end{aligned}$$

so they exhibit the same linear focus behaviour as the density $\rho (\mathbf{y} ,t|\mathbf{x} ,s)$ when approaching its steady state $P_0$. Therefore, we can extract the “stochastic asymptotic phase” $\psi (\mathbf {x})$ from $Q^*_{\lambda _1}(\mathbf {x})=u(\mathbf {x})e^{ i\psi (\mathbf {x})}$, so

18 $$\begin{aligned} \psi (\mathbf {x}) = \arg (Q^*_{\lambda _1}(\mathbf {x})), \end{aligned}$$

provided $u(\mathbf {x}) \ne 0$. Analogously to the deterministic case, we will define the points

19 $$\begin{aligned} {\bar{u}} = \{\mathbf {x}\mid u(\mathbf {x}) = 0 \}, \end{aligned}$$

in which a phase cannot be defined as “phaseless sets”.

The expected value of $\psi (\mathbf {x})$ follows (see “Appendix B” for the complete calculation details)

20 $$\begin{aligned} \frac{\mathrm{d}}{\mathrm{d}t} \mathbb {E}[\psi (\mathbf {x})] = \omega - 2\sum _{i,j} \mathcal {G}_{ij} \partial _i \ln (u(\mathbf {x})) \partial _j \psi (\mathbf {x}), \end{aligned}$$

hence, in the limit $\mathcal {G} \rightarrow 0$, the dynamics for $\mathbb {E}[\psi (\mathbf {x})]$ follow the dynamics for the deterministic phase Eq. (3), provided the deterministic system has a well-defined phase (see also “Appendix D” for a discussion about the relationship between $\psi (\mathbf {x})$ and $\vartheta (\mathbf {x})$ in the noise vanishing limit). Moreover, if the assumptions under which the uniqueness of solutions of Eq. (12) are met (see next Sect. 4 for a brief review of such conditions), from Eq. (20) it follows that if $\sum _{i,j} \mathcal {G}_{ij} \partial _i \ln (u(\mathbf {x})) \partial _j \psi (\mathbf {x})=0$, then $\overline{T}=2\pi /\omega$.

## Mathematical relation between the phases

Following Cao et al. (2020), we assume (without loss of generality) the existence of a parameterisation $\mathbf {x}=K(\alpha , \beta )$^1^

21 $$\begin{aligned} \begin{aligned} K : \mathbb {T} \times [R_-, R_+] \subset \mathbb {T} \times \mathbb {R}&\rightarrow \mathcal {R} \subset \mathbb {R}^2\\ (\alpha , \beta )&\rightarrow K(\alpha , \beta ) \end{aligned} \end{aligned}$$

such that the original domain $\mathcal {R} \subset \mathbb {R}^2$ of system Eq. (7) can be mapped to an annulus via an angular variable $\alpha (\mathbf {x}) \in [0, 2\pi )$ and an amplitude-like variable $\beta (\mathbf {x}) \in [R_-, R_+]$. Furthermore, we require the noise matrix $\mathcal {G}$ in Eq. (9) to be nondegenerate (invertible), so that the $\mathcal {L}^\dagger$ operator is strongly elliptic (McLean 2000). For the Fokker–Planck equation (9), we impose reflecting boundary conditions at $R_\pm$; for the backward equation (10) we impose adjoint reflecting boundary conditions. For the complete details on the assumptions required for the MRT theory to apply, see Cao et al. (2020).

With these assumptions, we next show how the MRT phase $\varTheta (\mathbf {x})$ can be represented as the stochastic asymptotic phase $\psi (\mathbf {x})$ plus an additional phase shift $\varDelta \psi (\mathbf {x})$. Consider the equality:

22 $$\begin{aligned} \mathcal {L}^\dagger [Q^*_{\lambda _1}(\mathbf {x})] = (\mu + i\omega )Q^*_{\lambda _1}(\mathbf {x}); \end{aligned}$$

using $Q^*_{\lambda _1}=u(\mathbf {x})e^{ i\psi (\mathbf {x})}$ and the definition of the $\mathcal {L}^\dagger$ operator, dividing by $e^{i\psi (\mathbf {x})}$, taking the imaginary part and assuming $u(\mathbf {x})$ is a non-vanishing function in $\mathcal {R}$, we find (see “Appendix B” for a complete derivation)

23 $$\begin{aligned} \mathcal {L}^\dagger [\psi (\mathbf {x})] + 2\sum _{i,j} \mathcal {G}_{ij} \partial _i \ln (u(\mathbf {x})) \partial _j \psi (\mathbf {x}) = \omega . \end{aligned}$$

Considering the difference between the two phases, $\varDelta \psi (\mathbf {x})=\varTheta (\mathbf {x}) -\psi (\mathbf {x}),$ it obeys the equation

24 $$\begin{aligned} \mathcal {L}^\dagger [\varDelta \psi (\mathbf {x})] = \overbrace{2\sum _{i,j} \mathcal {G}_{ij} \partial _i \ln (u(\mathbf {x})) \partial _j \psi (\mathbf {x})}^{\varOmega (\mathbf {x})}+ \overbrace{\frac{2\pi }{\overline{T}}-\omega }^{\varDelta \omega } \end{aligned}$$

Indeed, if we use $\varTheta (\mathbf {x})=\psi (\mathbf {x}) +\varDelta \psi (\mathbf {x})$, we obtain

25 $$\begin{aligned} \begin{aligned} \mathcal {L}^\dagger [\varTheta (\mathbf {x})]&= \mathcal {L}^\dagger [\psi (\mathbf {x}) + \varDelta \psi (\mathbf {x})] \\&= \mathcal {L}^\dagger [\psi (\mathbf {x})] + \mathcal {L}^\dagger [\varDelta \psi (\mathbf {x})] = \\&= \mathcal {L}^\dagger [\psi (\mathbf {x})] + \varOmega (\mathbf {x}) + \varDelta \omega = \frac{2 \pi }{\overline{T}}, \end{aligned} \end{aligned}$$

which is exactly the definition of MRT phase in Eq. (12).

As one can see, since both phase functions, $\varTheta (\mathbf {x})$ and $\psi (\mathbf {x})$, have a $2 \pi$ jump, the function $\varDelta \psi (\mathbf {x}) = \varTheta (\mathbf {x}) - \psi (\mathbf {x})$ will not have a jump. We notice the dependence of $\mathcal {L}^\dagger [\varDelta \psi (\mathbf {x})]$ on the term $\varDelta \omega$ corresponding to the difference between the frequency $\omega$ of the slowest decaying complex eigenmode $Q^*_{\lambda _1}(\mathbf {x})$ and the MRT frequency $2\pi /\overline{T}$. Whereas $\omega$ can be extracted from the spectra of $\mathcal {L}^\dagger$, the MRT period $\overline{T}$ can be obtained from the stationary probability current $\mathbf {J}_0$, which is given by

$$\begin{aligned} {\mathbf {J}}_0 = \begin{bmatrix} J_{0,x} \\ J_{0,y} \end{bmatrix} = \begin{bmatrix} f_x \\ f_y \end{bmatrix} P_0 - \frac{1}{2} \begin{bmatrix} \partial _{x}(\mathcal {G}_{xx}P_0) + \partial _{y}(\mathcal {G}_{xy}P_0)\\ \partial _{x}(\mathcal {G}_{yx}P_0) + \partial _{y}(\mathcal {G}_{yy}P_0) \end{bmatrix}, \end{aligned}$$

where $P_0(\mathbf {x})$ corresponds to the stationary probability density, satisfying $\mathcal {L}[P_0(\mathbf {x})] = 0$ and $\int P_0(\mathbf {x}) \mathrm{d}\mathbf {x}= 1$. As shown in Cao et al. (2020), one obtains the mean period by integrating the $\alpha$-component of the current $\mathbf {J}_0$ along a simple smooth, non-self-intersecting curve, connecting the inner and outer domain boundaries. That is,

26 $$\begin{aligned} \frac{1}{\overline{T}} = \int _{R_-}^{R_+} J_{0,\alpha }(\alpha , \beta ) \mathrm{d}\beta . \end{aligned}$$

See “Appendices A.2 and C” for details of the numerical calculation, and an analytical solvable example of Eq. (26), respectively.

In conclusion, solving for and using the first two functions of the eigenvalue problem for $\mathcal {L}$ and $\mathcal {L}^\dagger$ gives us both the stochastic asymptotic phase, the MRT phase, and the difference between the two phases.

## Examples

Next, we consider different examples to study how the MRT phase $\varTheta (\mathbf {x})$ and the stochastic asymptotic phase $\psi (\mathbf {x})$ are related. We will proceed under the assumption that all the models we study satisfy the eigenfunction expansion in Eq. (16), as well as the regularity assumptions given in Cao et al. (2020). For numerical details about computations in this Section, we refer the reader to the numerical “Appendix A”.

### Spiral sink

We start considering a classical and well-studied stochastic process: a two-dimensional Ornstein–Uhlenbeck process (OUP) in a setting such that the origin becomes a stable sink (Uhlenbeck and Ornstein 1930; Gardiner 1985; Leen et al. 2016; Thomas and Lindner 2019). The general Langevin equation is:

27 $$\begin{aligned} \dot{\mathbf {x}} = A\mathbf {x}+ B\xi \end{aligned}$$

where we assume that the two eigenvalues of *A* are a complex conjugate pair denoted as $\lambda _\pm = \mu \pm i \omega$ with $\mu <0$ and $\omega >0$. We write the matrices *A* and *B* as

28 $$\begin{aligned} A= & {} \begin{pmatrix} \mu &{} -\omega \\ \omega &{} \mu \end{pmatrix} ,\nonumber \\ B= & {} \begin{pmatrix} B_{11} &{} B_{12} \\ B_{21} &{} B_{22} \end{pmatrix}. \end{aligned}$$

For Eq. (27), the matrix $\mathcal {G} = \frac{1}{2} BB^\top$ in $\mathcal {L}^\dagger$ (see Eq. (9)) can be written in the following way

29 $$\begin{aligned} \begin{aligned} \mathcal {G}&= \frac{1}{2} \begin{pmatrix} B^2_{11} + B^2_{12} &{} B_{11}B_{21} + B_{12}B_{22} \\ B_{11}B_{21} + B_{12}B_{22} &{} B^2_{22} + B^2_{21} \end{pmatrix}\\\\&= \epsilon \begin{pmatrix} 1 + \beta _D &{} \beta _c \\ \beta _c &{} 1 - \beta _D \end{pmatrix} \end{aligned} \end{aligned}$$

Following Thomas and Lindner (2019), we know the asymptotic phase function for Eq. (27) is written as^2^

30 $$\begin{aligned} \psi (\mathbf {x}) = \arctan (\mathbf {x}_2/\mathbf {x}_1) \end{aligned}$$

whose expected value follows

31 $$\begin{aligned} \begin{aligned} \frac{\mathrm{d}}{\mathrm{d}t} \mathbb {E}[\psi (\mathbf {x})]&= \omega + 4\epsilon \left( \frac{\beta _D \mathbf {x}_1\mathbf {x}_2}{(\mathbf {x}_1^2 + \mathbf {x}_2^2)^2} - \frac{\beta _c(\mathbf {x}_1^2 - \mathbf {x}_2^2)}{2(\mathbf {x}_1^2 + \mathbf {x}_2^2)^2} \right) \\&= \omega - \varOmega (\mathbf {x}), \end{aligned} \end{aligned}$$

showing that, as long as there is some noise in the system ($\epsilon > 0$), the term $\varOmega (\mathbf {x})$ diverges at the origin.

In “Appendix C”, we derive the following expression for the mean period $\overline{T}$:

32 $$\begin{aligned} \overline{T}= \frac{2 \pi (\omega ^2 + \mu ^2(1 - \beta ^2_c - \beta ^2_D))}{\omega (\mu ^2 + \omega ^2)}. \end{aligned}$$

which, together with Eq. (31) yields that if $\beta _D = \beta _c = 0$, that is for isotropic noise of the same amplitude, the MRT phase and the stochastic asymptotic phase for the stochastic sink in Eq. (27) are equivalent.

**Fig. 1:**
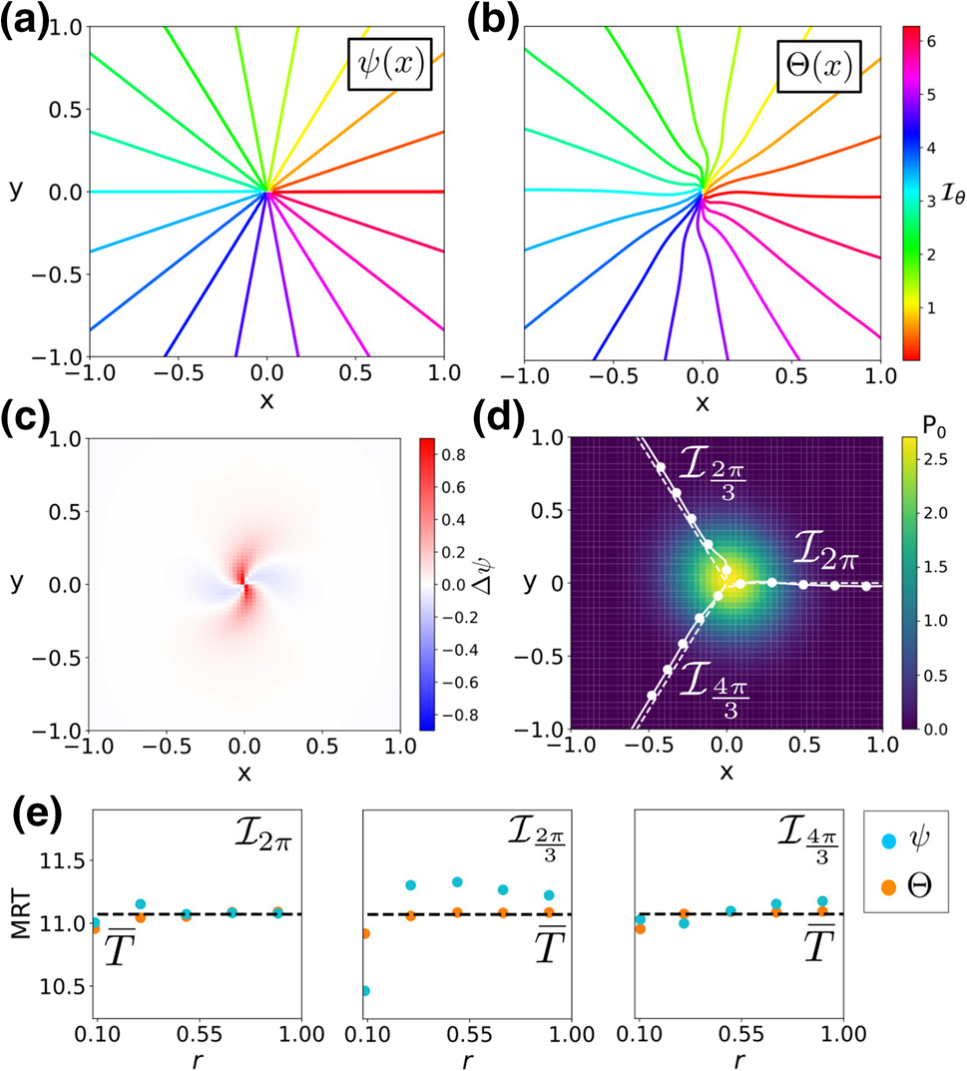
Phase analysis of a noisy linear focus Eq. (27). **a**, **b** Level curves of the stochastic asymptotic phase $\psi (\mathbf {x})$ and the MRT $\varTheta (\mathbf {x})$ ($\mathbf {y}$-label shared). **c** Difference $\varDelta \psi$ **d** Stationary probability distribution (colour coded), with a comparison between some level sets of $\varPsi (\mathbf {x})$ (dashed) and $\varTheta (\mathbf {x})$ (solid). **e** Comparison of the MRT property ($\overline{T}\approx 11.07, 2\pi /\omega \approx 11.13$) for $\psi (\mathbf {x})$ (blue) and $\varTheta (\mathbf {x})$ (orange) for three different MRT-isochrons.

To illustrate the OUP case, we choose coefficients from Powanwe and Longtin (2019), in which a noisy focus is used to model fast gamma-band brain signals (see also Duchet et al. (2020); Spyropoulos et al. (2020) for similar modelling approaches in a neuroscience context). In particular, we take $A=[0.1598$, $-0.52;$ 0.7227,  $-0.319]$ and $B=\sqrt{2D}\cdot [1,0;0,0.5]$ with $D=0.01125$. After a linear change of variables, we put the model in the form of Eq. (28), with $\mu = -0.0796, \omega = 0.564$ and $B = [0, 0.0492, -0.126, 0.0214]$ from which we obtain $\epsilon , \beta _D,$ $\beta _c = [0.0046, -0.74, 0.11]$.

Figure 1a, b shows both level curves of the stochastic asymptotic phase $\psi (\mathbf {x})$ and the MRT phase $\varTheta (\mathbf {x})$. As Eq. (31) indicates, the farther we move from the origin, the smaller the term $\varOmega (\mathbf {x})$ becomes. As a consequence, the difference $\varDelta \psi (\mathbf {x})$ is almost negligible far from the origin (panel c) thus causing $\psi (\mathbf {x})$ and $\varTheta (\mathbf {x})$ to differ just in a small neighbourhood of the origin (panel d). In this case, thanks to the analytical expression for $\varDelta \psi$ (see “Appendix A”), we are able to find the values of the difference near the phaseless set (the origin). As Fig. 1c illustrates, near the origin, the term $\varDelta \psi (\mathbf {x})$ alternates between positive and negative values. Panel (e) confirms that the resulting MRT isochrons have the MRT property. While the numerically computed MRT isochrons satisfy the MRT property with high accuracy, the isochrons based on the stochastic asymptotic phase show small but significant deviations from uniformity.

### Noisy Wilson–Cowan

Next, we study a noisy version of the Wilson–Cowan (WC) equations, which are widely used to model large-scale neural activity (Wilson and Cowan 1973; Destexhe and Sejnowski 2009; Akam et al. 2012). We adopt the form

33 $$\begin{aligned} \begin{aligned} {\dot{E}}&= -E + S_e(c_1E - c_2I + P) + D_e \xi _e(t),\\ {\dot{I}}&= -I + S_i(c_3E - c_4I + Q) + D_i \xi _i(t), \end{aligned} \end{aligned}$$

with $S_{e,i}(x) = \left[ 1+\exp \left( -a_{e,i}(x-\theta _{e,i})\right) \right] ^{-1}$ being the sigmoidal activation function and $\xi _{e,i}(t)$ being Gaussian white noise. Here, $D_e, D_i = D\cdot [1, 0.5]$ with $D = 0.1$. Since the conditions for the WC model to show oscillations are well known (Wilson and Cowan 1972; Borisyuk and Kirillov 1992), we choose parameters $c_{1}$, $c_{2}$, $c_{3}$, $c_{4}$, $a_e$, $a_i$, $\theta _e$, $\theta _i$ = [13, 12, 6, 4, 1.3, 2, 4, 1.5] and use *P*, *Q* as bifurcation parameters. We choose $(P, Q) = (2.5, 0)$, so the system Eq. (33) shows a stable limit cycle of period $T=5.26$ (Pérez-Cervera et al. 2020b).

**Fig. 2:**
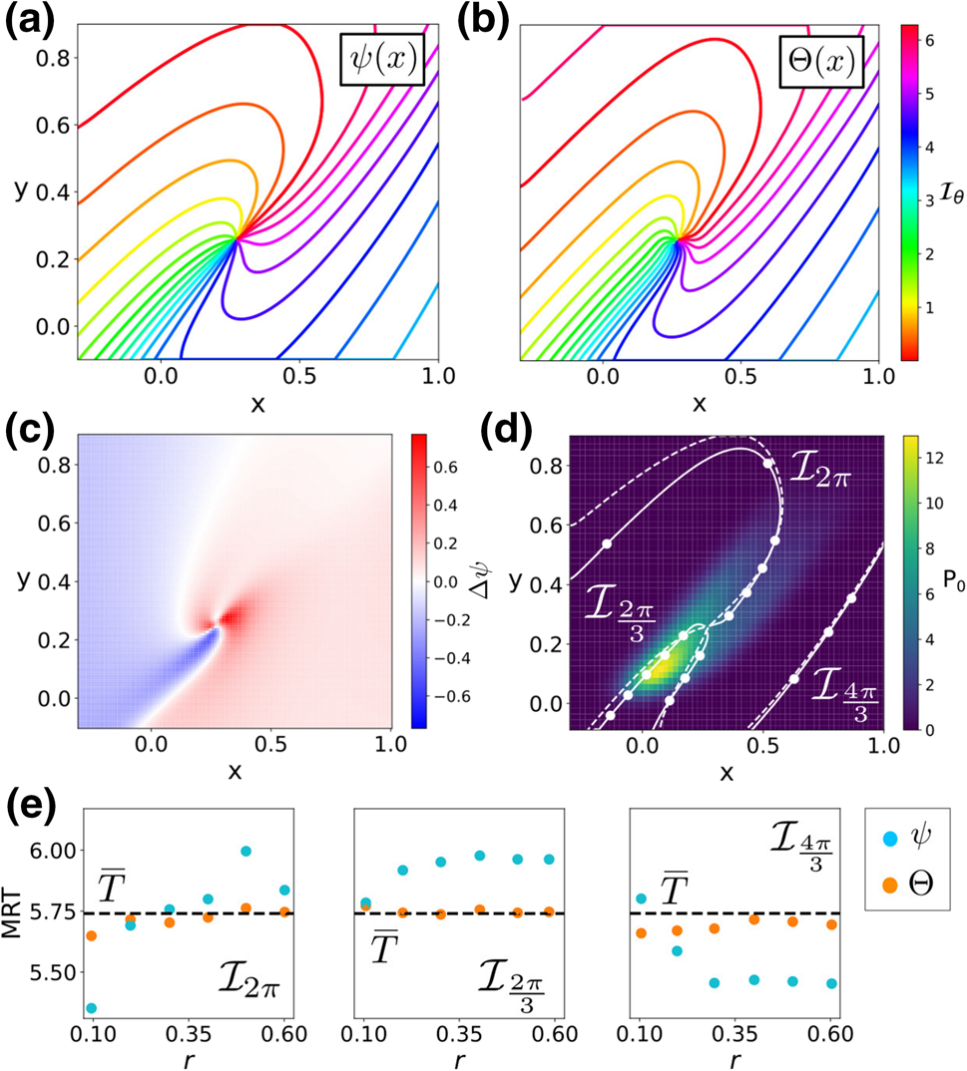
Phase analysis of a noisy Wilson–Cowan system near a Hopf bifurcation. **a**, **b** Level curves of the stochastic asymptotic phase $\psi (\mathbf {x})$ and the MRT phase $\varTheta (\mathbf {x})$ ($\mathbf {y}$-label shared). **c** Phase difference $\varDelta \psi$ **d** Stationary probability distribution (colour coded), with a comparison between level sets of $\psi (\mathbf {x})$ (dashed) and $\varTheta (\mathbf {x})$ (solid). **e** Comparison of the MRT property ($\overline{T}\approx 5.7$, $2\pi /\omega \approx 5.99$) for $\psi (\mathbf {x})$ (blue) and $\varTheta (\mathbf {x})$ (orange) for three different MRT-isochrons

Figure 2 displays the results. Panels (a, b) compare the level curves of the stochastic asymptotic phase $\psi (\mathbf {x})$ and the MRT phase $\varTheta (\mathbf {x})$. In this case, the differences between both level curves are more striking than in the linear focus case. As Fig. 2c shows, and similarly as in the linear focus case, near the phaseless set of the deterministic system, the difference $\varDelta \psi$ alternates between positive and negative values. However, the nonlinearities of Eq. (33) cause $\varDelta \psi$ to differ from 0 in the bulk of the domain, thus causing differences between the level sets of $\psi (\mathbf {x})$ and $\varTheta (\mathbf {x})$ (see panel d). Panel (e) demonstrates that the isochrons of the numerically computed MRT phase $\varTheta (\mathbf {x})$ satisfy the MRT property to within a 1% margin of error, while the isochrons of the stochastic asymptotic phase $\psi (\mathbf {x})$ do not. Moreover, panel (e) also shows that the larger the differences between the level curves of $\psi (\mathbf {x})$ and $\varTheta (\mathbf {x})$, the larger the deviations of $\psi (\mathbf {x})$ from the MRT property (compare results in panel (e) for $\mathcal {I}_{2 \pi }$ and $\mathcal {I}_{\frac{4 \pi }{3}}$). This result confirms our expectations.

**Fig. 3:**
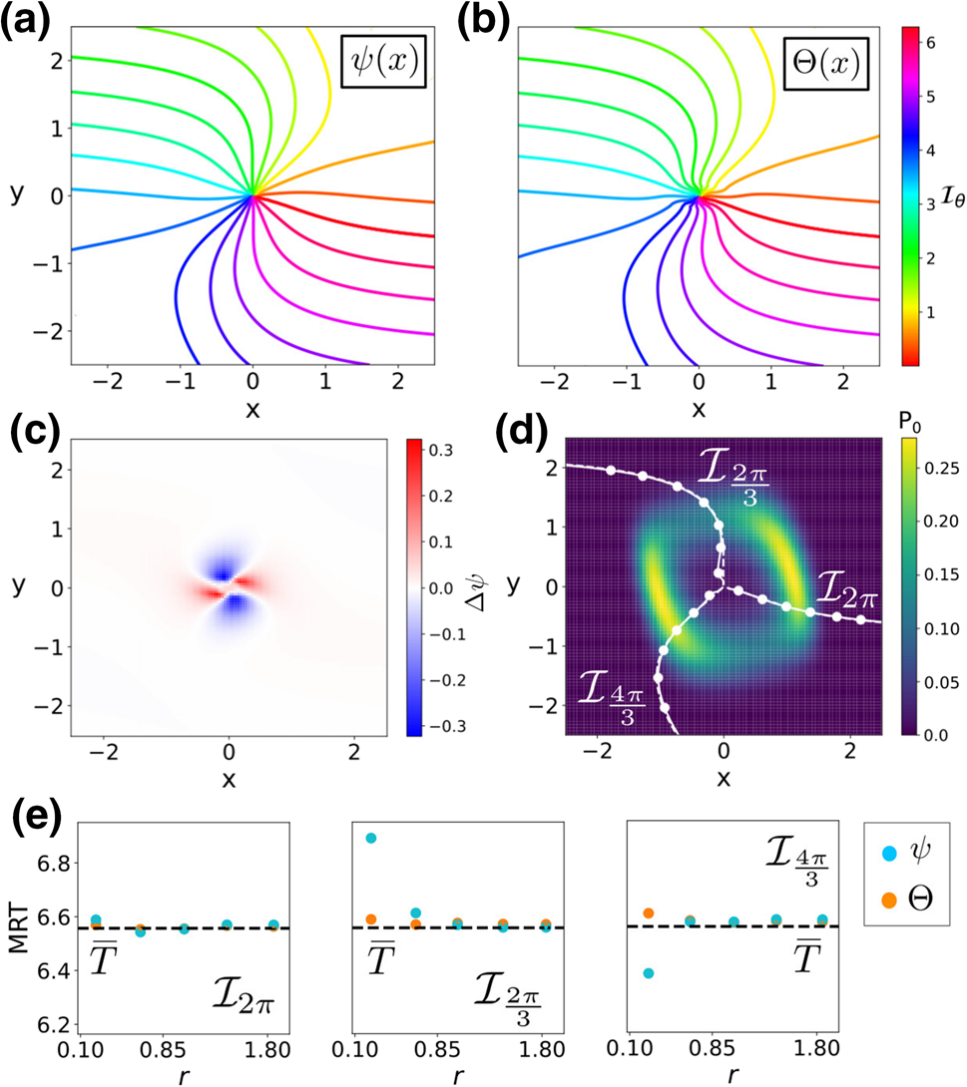
Phase analysis of a noisy Van der Pol oscillator with anisotropic noise $[D_x, D_y] = \sqrt{2D}\cdot [1, 0.1]$ with $D=0.1$. **a**, **b** Level curves of the stochastic asymptotic phase $\psi (\mathbf {x})$ and the MRT phase $\varTheta (\mathbf {x})$ ($\mathbf {y}$-label shared). **c** Phase difference $\varDelta \psi$. **d** Stationary probability distribution (colour coded), with a comparison between level sets of $\psi (\mathbf {x})$ (dashed) and $\varTheta (\mathbf {x})$ (solid). **e** Comparison of the MRT property ($\overline{T}\approx 6.55$, $2\pi /\omega \approx 6.59$) for $\psi (\mathbf {x})$ (blue) and $\varTheta (\mathbf {x})$ (orange) for three different MRT-isochrons

### Noisy Van der Pol oscillator

Next, we study a stochastic version of the Van der Pol equations,

34 $$\begin{aligned} \begin{aligned} {\dot{x}}&= -y + x - x^3 + D_x \xi _x(t),\\ {\dot{y}}&= x + D_y \xi _y(t), \end{aligned} \end{aligned}$$

which in the absence of noise displays a limit cycle of period $T \approx 6.663$. In this case, we set the noise in each component to be $[D_x, D_y] = \sqrt{2D}\cdot [1, 0.1]$, with $D=0.1$. Due to their similarity with the FitzHugh–Nagumo model (FitzHugh 1961; Nagumo et al. 1962), these equations are widely used in neuroscience as a useful reduction in the Hodgkin–Huxley neuron model (Izhikevich 2007). At a macroscopic level, they are also used to describe successfully the dynamics of epileptic tissue (Proix et al. 2017; Pérez-Cervera and Hlinka 2021).

We illustrate results for this oscillator in Fig. 3. As panels (a, b) illustrate, differences between the stochastic asymptotic phase $\psi (\mathbf {x})$ and the MRT phase $\varTheta (\mathbf {x})$ are very small and they are restricted to a neighbourhood of the origin. Indeed, the structure of the discrepancies is similar to the differences for the OUP. That is, they alternate between negative and positive values (compare panel (c) in Figs. 1, 3). We observe (panel d) that both phases are almost identical near the maxima of the stationary distribution $P_0$. Together with the near equivalence of both periods $2\pi /\omega \approx 6.59$, $\overline{T}\approx 6.55$, their similarity causes both phases to be nearly indistinguishable in this case. Indeed, as panel (e) shows, the MRT property is satisfied very accurately for both phases, except for $\psi (\mathbf {x})$ in points very near the origin (where phase discrepancies $\varDelta \psi (\mathbf {x})$ are larger).

### Noisy heteroclinic oscillator

We complete our comparison between the stochastic asymptotic phase $\psi (\mathbf {x})$ and the MRT phase $\varTheta (\mathbf {x})$ by studying a noisy heteroclinic oscillator. The specific form of the deterministic heteroclinic system we consider was introduced in Hirsch et al. (2012), chapter 10, and was adapted to a biological context as a conceptual model for a central pattern generator control mechanism based on a dynamical architecture alternative to the standard limit cycle architecture (Shaw et al. 2012, 2015; Lyttle et al. 2017; Park et al. 2018). We study the form given in (Giner-Baldo et al. 2017), namely

35 $$\begin{aligned} \begin{aligned} \dot{\mathbf {X}}&= \cos (\mathbf {X})\sin (\mathbf {Y}) + \alpha \sin (2 \mathbf {X}) + \sqrt{2 D}\xi _1(t) \\ \dot{\mathbf {Y}}&= -\sin (\mathbf {X})\cos (\mathbf {Y}) + \alpha \sin (2 \mathbf {Y}) + \sqrt{2 D}\xi _2(t) \end{aligned} \end{aligned}$$

with $\alpha = 0.1, D = 0.01125$ and reflecting boundary conditions on the domain $-\pi /2 \le \{\mathbf {X}, \mathbf {Y}\} \le \pi /2$. Without noise, Eq. (35) has an attracting heteroclinic cycle, consisting of a closed loop of trajectories connecting a sequence of saddle equilibria which is capable of sustaining robust oscillations in the presence of noise (Thomas and Lindner 2014).

**Fig. 4:**
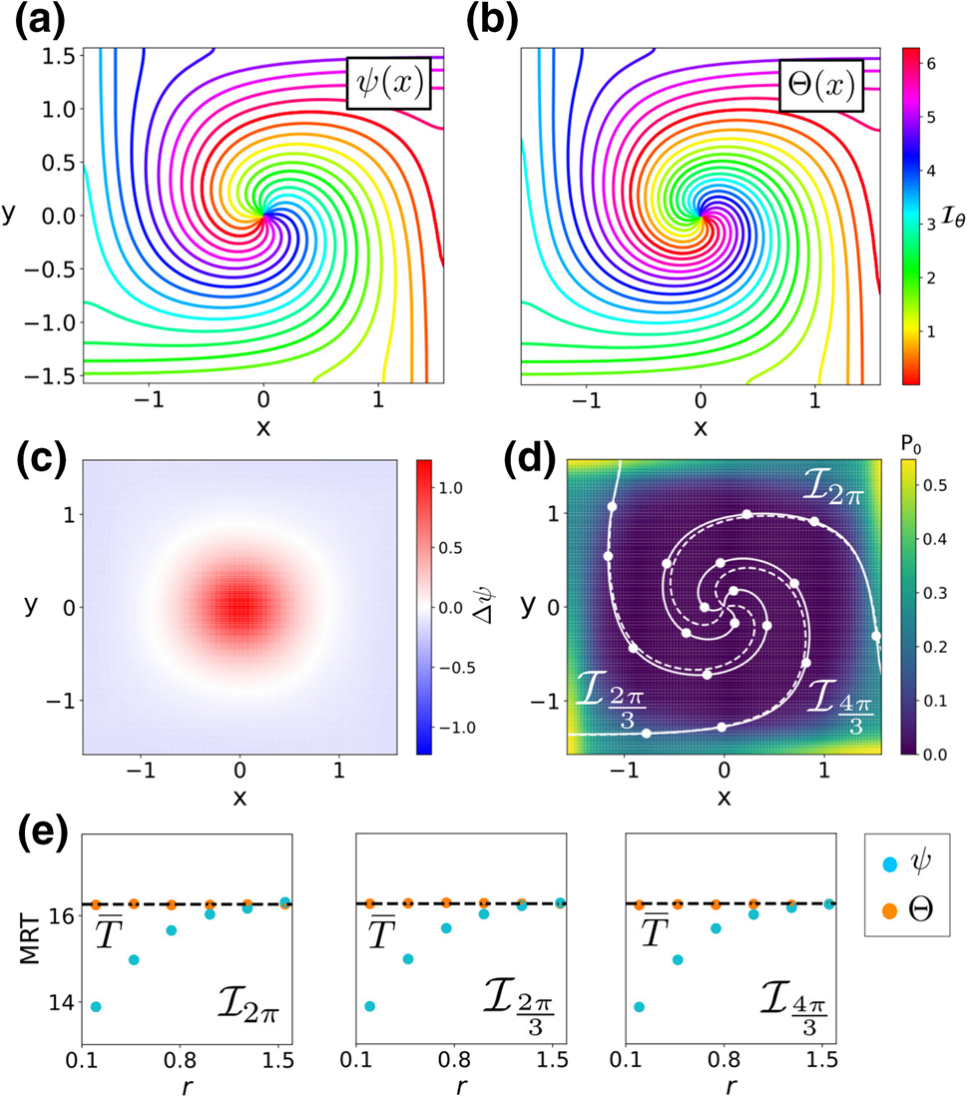
Phase analysis of a noisy Heteroclinic Oscillator with lower ($D = 0.01125$) noise. **a**, **b** Level curves of the stochastic asymptotic phase $\psi (\mathbf {x})$ and the MRT phase $\varTheta (\mathbf {x})$ ($\mathbf {y}$-label shared). **c** Phase difference $\varDelta \psi$ **d** Stationary probability distribution (colour coded), with a comparison between some level sets of $\psi (\mathbf {x})$ (dashed) and $\varTheta (\mathbf {x})$ (solid). **e** Comparison of the MRT property ($\overline{T}\approx 16.26, 2\pi /\omega \approx 16.38$) for $\psi (\mathbf {x})$ (blue) and $\varTheta (\mathbf {x})$ (orange) for three different MRT-isochrons

**Fig. 5:**
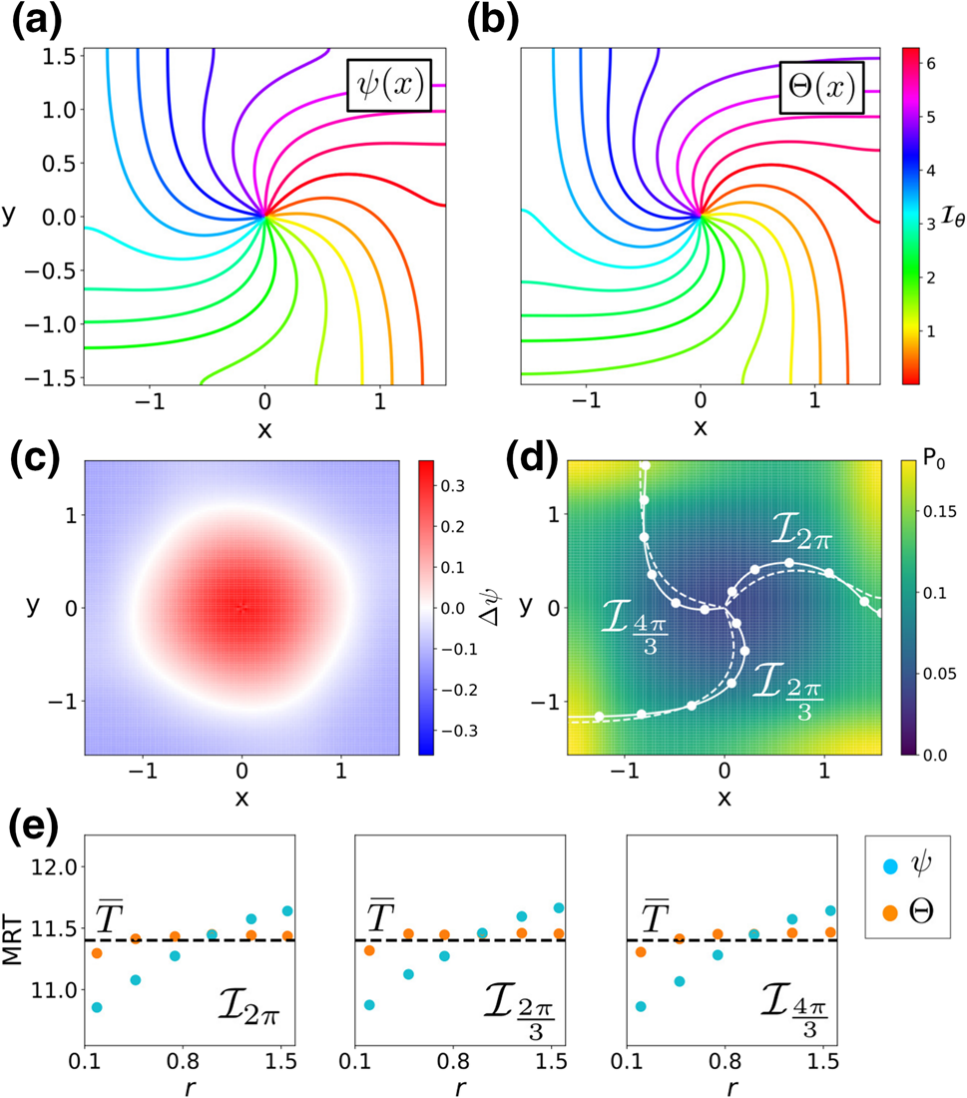
Phase analysis of a noisy Heteroclinic Oscillator with higher ($D = 0.1$) noise. **a**, **b** Level curves of the stochastic asymptotic phase $\psi (\mathbf {x})$ and the MRT phase $\varTheta (\mathbf {x})$. **c** Phase difference $\varDelta \psi$ **d** Stationary probability distribution (colour coded), with a comparison between some level sets of $\psi (\mathbf {x})$ (dashed) and $\varTheta (\mathbf {x})$ (solid) ($\mathbf {y}$-label shared). **e** Comparison of the MRT property ($\overline{T}\approx 11.41, 2\pi /\omega \approx 12.43$) for $\psi (\mathbf {x})$ (blue) and $\varTheta (\mathbf {x})$ (orange) for three different MRT-isochrons

We study this oscillator for lower ($D=0.01125$) and higher ($D=0.1$) level of noise and present results for each case in Figs. 4 and 5, respectively. Panels (a, b) compare the stochastic asymptotic phase $\psi (\mathbf {x})$ and the MRT phase $\varTheta (\mathbf {x})$. In contrast to the previous cases, the structure of the phase difference $\varDelta \psi (\mathbf {x})$ appears to be rotationally symmetric, to a good approximation, in a neighbourhood of the centre (see both c panels). These differences in phase lead to the principal discrepancies between the level curves of both phases appearing near the origin (panel d). Indeed, as the MRT property check in panel (e) shows, whereas the MRT property holds quite well for points computed using the MRT phase $\varTheta (\mathbf {x})$, it does not hold in general for the stochastic asymptotic phase $\psi (\mathbf {x})$. However, for lower noise, the MRT property holds for points away from the origin in which the level curves for both phases coincide and the stationary probability is concentrated. As noise increases, the mean return time of the stochastic asymptotic phase becomes increasingly position-dependent close to the domain border.

## Discussion

In this paper, we have derived a framework to compute simultaneously the MRT phase and the asymptotic phase of a stochastic oscillator. Our results build on prior work by Schwabedal and Pikovsky (2013) and Thomas and Lindner (2014) defining the notions of MRT phase $\varTheta (\mathbf {x})$ and stochastic asymptotic phase $\psi (\mathbf {x})$, respectively. While initially defined on an algorithmic basis, the MRT phase was recast by Cao et al. (2020) as the solution of a PDE with jump-periodic boundary conditions. As a result of Cao et al. (2020), a relationship between the Kolmogorov backwards $\mathcal {L}^\dagger$ operator and the MRT phase was derived (see Eq. (12)). Since the stochastic asymptotic phase was already defined as the argument of the slowest decaying eigenfunction of $\mathcal {L}^\dagger$, in this work we developed the link between both phases and the Kolmogorov backwards operator to obtain an expression for the difference between the two phases. That is, $\varTheta (\mathbf {x}) = \psi (\mathbf {x}) + \varDelta \psi (\mathbf {x})$, with $\varDelta \psi (\mathbf {x})$ satisfying Eq. (24).

The computation of the difference $\varDelta \psi (\mathbf {x})$ allowed us to compare both phases in different dynamical scenarios: we have considered two examples of noise induced oscillations (a spiral sink and an heteroclinic oscillator) and noisy limit cycle dynamics. Formally, from the uniqueness results in Cao et al. (2020) (see Theorem 3.1) it follows that both phases are not equivalent if the term $\varOmega (\mathbf {x}) = \sum _{i,j} \mathcal {G}_{ij} \partial _i \ln (u(\mathbf {x})) \partial _j \psi (\mathbf {x})$ in the right hand side of Eq. (20) is not zero. As Cao (2017) observed, for a planar oscillator with isotropic noise, the condition $\varOmega (\mathbf {x})=0$ is satisfied if the eigenfunction $Q^*_{\lambda _1}$ is a complex analytic function of its arguments (in the sense of complex variables theory). But the practical significance of the difference $\varDelta \psi (\mathbf {x})$ has not been systematically explored before now.

### Two perspectives on stochastic oscillators

Anderson et al. (2015) articulate the distinction between *pathwise* and *ensemble* descriptions of a stochastic process. For example, a general diffusion process may be described either in terms of the Itô stochastic differential equation

$$\begin{aligned} \dot{\mathbf {X}}=\mathbf {f}(\mathbf {X})+\mathbf {g}(\mathbf {X})\xi (t), \end{aligned}$$

which describes the evolution of a single trajectory along a sample path, or in terms of the Fokker–Planck equation

$$\begin{aligned} \frac{\partial \rho }{\partial t}=-\nabla \left( \rho \mathbf {f}(\mathbf {x})\right) +\frac{1}{2}\sum _{ij}\frac{\partial ^2}{\partial x_i x_j}\left[ \left( \mathbf {g}\mathbf {g}^\intercal \right) _{ij}\rho \right] \end{aligned}$$

which describes the evolution of the density $\rho (\mathbf {x},t)=\frac{1}{|\mathrm d\mathbf {x}|}\text {Pr}\left[ \mathbf {X}(t)\in [\mathbf {x},\mathbf {x}+\mathrm{d}\mathbf {x})\right]$ of an ensemble of trajectories (Øksendal 2003).

Similarly, a discrete chemical reaction process comprising *M* reactions with stoichiometry vectors $\zeta _k$ and hazard functions $\lambda _k$, driven by independent Poisson processes $\left\{ Y_k(t)\right\} _{k=1}^M$, has a pathwise description of the form

$$\begin{aligned} \mathbf {X}(t)=\mathbf {X}_0+\sum _{k=1}^M \zeta _k Y_k\left( \int _0^t\lambda _k(\mathbf {X}(s))\,\mathrm{d}s\right) , \end{aligned}$$

as well as an evolution equation (the so-called chemical master equation (Higham 2008)) of the form

$$\begin{aligned} \frac{\mathrm{d}}{\mathrm{d}t}p(\mathbf {x},t)=\sum _{k=1}^M p(\mathbf {x}-\zeta _k,t)\lambda _k(\mathbf {x}-\zeta _k) - p(\mathbf {x},t)\sum _{k=1}^M\lambda _k(\mathbf {x}) \end{aligned}$$

where $p(\mathbf {x},t)$ is the probability that the state $\mathbf {X}(t)$ is exactly $\mathbf {x}$ at time *t* (Anderson and Kurtz 2015).

The transit time for a stochastic oscillator to reach a Poincaré section from a starting point on that section, having completed one full rotation, is a stopping time (Karatzas and Shreve 2012), thus, a random variable that arises from the individual sample path. The “mean–return-time” function $T(\mathbf {x})$ (Cao et al. 2020) is defined from the ensemble average of this quantity. Importantly, the MRT property describes the behaviour of trajectories over a finite time horizon, namely looking roughly one period into the future.

In contrast, one defines the stochastic asymptotic phase $\psi (\mathbf {x})$ (Thomas and Lindner 2014) in terms of the long-time statistical behaviour of an ensemble of trajectories, as captured by the biorthogonal eigenfunction expansion Eq. (15) of the forward and backward operators. Thus, while the equations satisfied by both the MRT function, namely $\mathcal {L}^\dagger [T]=-1$, and the stochastic asymptotic phase eigenfunction, namely $\mathcal {L}^\dagger [Q]=\lambda Q$, involve the backward Kolmogorov operator, we see the MRT as related to a “pathwise” description over a finite time horizon, and the asymptotic phase as related to the “ensemble” description of the process at long times.

We speculate that this distinction may turn out to play a role in choosing which notion of phase applies more naturally to specific problems, such as synchronization of coupled stochastic oscillators (long-time behaviour), or “phase response” of a stochastic oscillator to a single kick (short-time behaviour). Fortunately, as we have seen above, for many biological examples the quantitative difference between the two types of phase is small. Their quantitative similarity thus provides investigators a degree of flexibility in working with whichever notion of phase is conceptually best suited to a given problem.

### Noise amplitude

For systems with an underlying limit cycle, we observe empirically that both phases appear to coincide with the deterministic phase as the level of noise approaches zero. For the MRT phase, its convergence to the deterministic phase in the case of vanishing noise has been investigated by Cao et al. (2020) $\S$2.4, who established convergence under additional regularity assumptions. For the stochastic asymptotic phase, its convergence to the deterministic phase for vanishing noise was anticipated by Thomas and Lindner (2014), see also the discussion in terms of the Koopman operator by Kato et al. (2021) and derived in “Appendix D” in this manuscript using the same additional regularity assumptions as in Cao et al. (2020). In line with these observations, although the intrinsic differences between both phases depend on each particular system, we have found the differences between the MRT phase and the stochastic asymptotic phase to grow as the noise is increased. It is nevertheless interesting to observe that, in every case we have explored, the differences between both phase level curves were restricted to areas where the stationary density was low. Therefore, once differences in mean period were accounted for, both phases were practically indistinguishable when describing single trajectories, differing only in how well they satisfy the MRT property, which is a property of the ensemble.

**Fig. 6:**
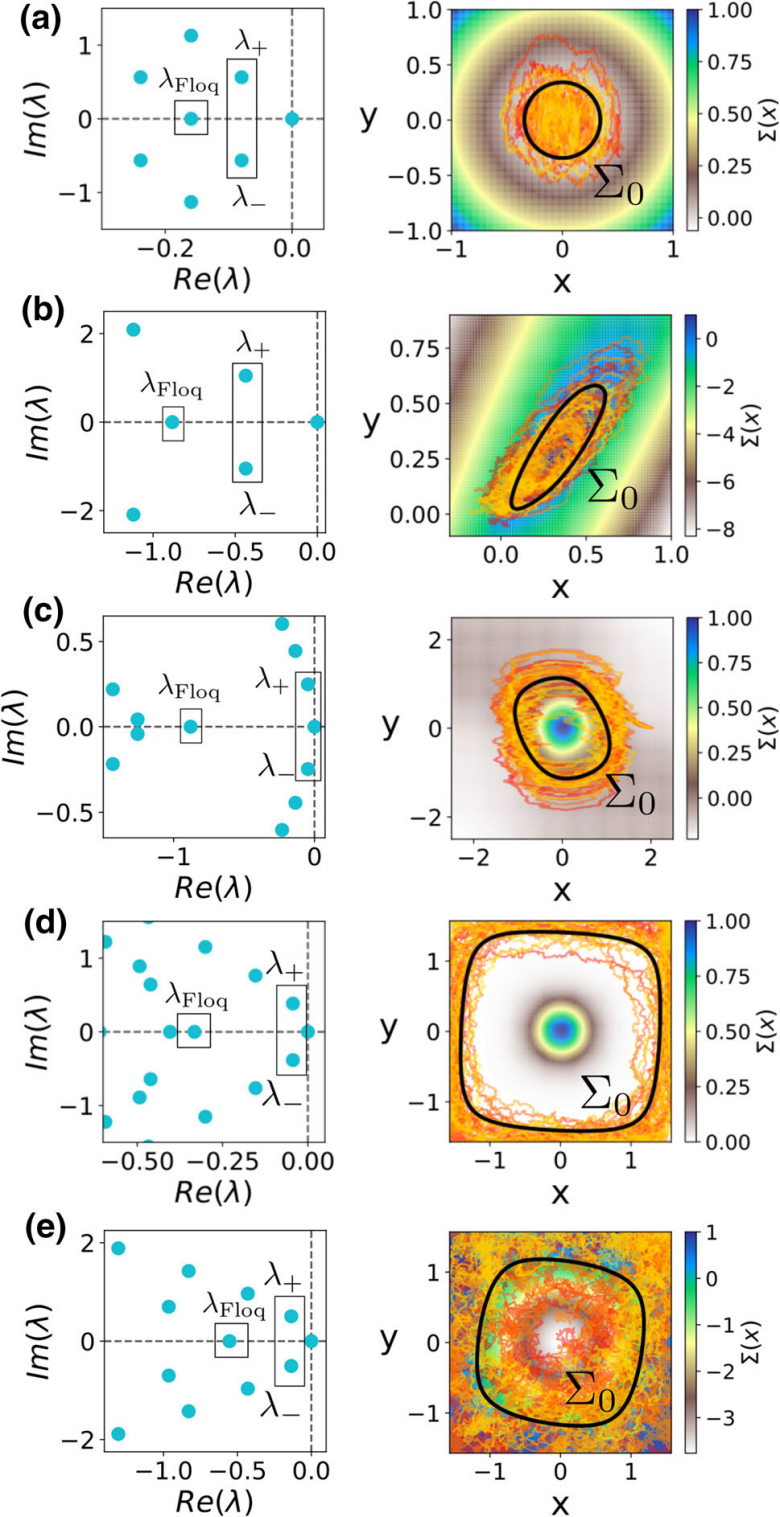
For the five models considered in the main text, namely, **a** the spiral sink in Eq. (27), **b** the noisy Wilson–Cowan in Eq. (33) and **c** the Van der Pol equations in Eq. (34) the noisy heteroclinic oscillator in Eq. (35) for lower **d** and larger **e** noise we show: Eigenvalue spectra of $\mathcal {L}^\dagger$ (left panel). The stochastic asymptotic phase $\psi (\mathbf {x})$ can be obtained from the argument of the eigenfunctions $Q^*_{\lambda _1}(\mathbf {x})$ associated with the eigenvalues the smallest non-negative purely *complex* eigenvalues $\lambda _\pm$ ($\lambda _+ = \lambda _1$). In addition, the eigenfunction $\varSigma (\mathbf {x})$ associated with the smallest non-negative purely *real* eigenvalue $\lambda _{\text {Floq}}$ corresponds to the stochastic isostables. Right panel: level curves of the isostable function $\varSigma (\mathbf {x})$, ten trajectories and the stochastic limit cycle $\varSigma _0\equiv \{\mathbf {x}\,|\,\varSigma (\mathbf {x})=0\}$ (black curve)

For a deterministic limit cycle (LC) system, in which both phase perspectives coincide, the function $e^{i\psi (\mathbf {x})}$ is an eigenfunction of $\mathcal {L}^\dagger$ with eigenvalue $\lambda _1 = i\omega$. As soon as some noise is introduced in the system $(\mathcal {G} \ne 0)$, the eigenvalue $\lambda _1$ associated with the slowest decaying eigenfunction becomes complex instead of purely imaginary, that is $\lambda _1 = \mu + i\omega$ (with $\mu < 0$). As a consequence, for $\mathcal {G} \ne 0$, the information about the initial phase is dissipated as time progresses. By contrast, we can think of the MRT function as a function containing information about the oscillatory system which *does not vanish* as $t \rightarrow \infty$. Therefore, for systems having an underlying LC, one can expect that the more robustly oscillatory the system (i.e. $|\mu | \ll \omega )$, the more similar $\psi (\mathbf {x})$ and $\varTheta (\mathbf {x})$ become. This interpretation agrees for the LC systems we studied: the Wilson–Cowan (WC) equations and Van der Pol (VdP) model. For the WC equations, we observed larger discrepancies between $\psi (\mathbf {x})$ and $\varTheta (\mathbf {x})$ than in the VdP model. These differences cause a loss of the MRT property for the stochastic phase, which is consistent with the magnitude of $|\mu /\omega |$ for both cases: $|\mu /\omega | \approx 0.415$ for the WC equations and $|\mu /\omega | \approx 0.054$ the VdP model, respectively (see Table 1 and Fig. 6 at “Appendix A”).

Despite not having a limit cycle, we observe that for the noisy heteroclinic oscillator (HO), the system is less robustly oscillatory as the noise increases. More precisely, $|\mu /\omega | \approx 0.115$ for $D= 0.01125$, and $|\mu /\omega | \approx 0.269$ for $D =0.1$, respectively (see Table 1 and Fig. 6). Indeed, our computations for the HO suggest that both $\mu$ and $\omega$ tend to 0 as $\mathcal {G} \rightarrow 0$, which can be interpreted as the system approaching an infinite period closed loop as $\mathcal {G} \rightarrow 0$. Hence, the results for the HO can be considered a particular case of the LC case, thus supporting the interpretation of the role of $|\mu /\omega |$ in the loss of the MRT property for the stochastic asymptotic phase.

The focus case requires a different interpretation. Unlike the LC or HO cases, stable focus systems have no closed loop structure in the absence of noise. Indeed, in the focus case $\mu$, approaches the negative nonzero real part of the stable focus eigenvalue as $\mathcal {G} \rightarrow 0$. As a consequence, the addition of noise may, in general, increases or decreases $|\mu /\omega |$. In this paper, we considered a widely used linear model: the Ornstein–Uhlenbeck process (OUP). For this model, we provided a new formula $\overline{T}$ and an initial seed for computing $\varDelta \psi (\mathbf {x})$ accurately even near the origin, thus facilitating the computation of its MRT function. However, the OUP system we studied has the particular property of not changing $|\mu /\omega |$ as the noise increases. Studying how the change of $\mu /\omega$ affects the phase dynamics and other important features of nonlinear stochastic foci, such as the amplitude of the stochastic oscillator (Pérez-Cervera et al. 2021), appears as an interesting topic for further research.

### Future perspectives

In developing our numerical examples, we have proceeded under the assumption that the robustly oscillatory criteria are met and that each system studied has a complete biorthogonal eigenfunction expansion. Whether this is rigorously true or not in specific cases is a question of functional analysis that goes beyond the scope of this paper. However, although our theoretical development assumes the expansion, it appears that practically, all that is really required is that the low-lying spectral elements (eigenmodes with eigenvalues having relatively small real and imaginary parts) exist and are discrete.

From a numerical perspective, the methodology introduced in this paper extends the numerical procedure introduced in Cao et al. (2020) which assumes the location of the phaseless set to be known a priori (see Section A.4 in the “Appendix” for further discussion of this point). However, whereas our procedure is restricted to systems in which the set of SDEs describing the system is known, and the noise is temporally uncorrelated and Gaussian, the procedure presented in Schwabedal and Pikovsky (2013) (based on Monte Carlo simulations) applies to a wide range of systems, noise types and also to data. Nevertheless, due to the equivalence between the Kolmogorov backwards operator and the stochastic Koopman operator (Črnjarić-Žic et al. 2019), we expect that the computation of the eigenfunctions of interest from data via dynamical decomposition methods (Schmid 2010; Budišić et al. 2012; Proctor et al. 2016; Brunton and Kutz 2019; Mauroy et al. 2020), combined with the theoretical expression we gave in Eq. (24), may provide an alternative way of obtaining the MRT phase from data.

## A Numerical details

In this Appendix, we detail the numerical methodology leading to the results in this paper. Results in this Appendix follow from previous work in Cao et al. (2020); Pérez-Cervera et al. (2021).

### A.1 Diagonalization of the $\mathcal {L}^\dagger$ operator

We construct the $\mathcal {L}^\dagger$ operator as follows.

Given a Langevin equation as in Eq. (7), we restrict its phase space to a rectangular domain

36 $$\begin{aligned} \mathcal {D} = [ x^-, x^+ ] \times [y^-, y^+]. \end{aligned}$$

whose size is chosen large enough so that the probability for individual trajectories $\mathbf {X}(t)$ to reach the boundaries is very low. For the special case of the heteroclinic oscillator, boundaries are given by the nature of the system. Then, we discretize the domain $\mathcal {D}$ in *N* and *M* points such that $\varDelta x = (x_+ - x_-)/N$ and $\varDelta y = (y_+ - y_-)/M$. Then, we build $\mathcal {L}^\dagger$ by using a standard centred finite difference scheme, except at the borders of the domain. For the heteroclinic oscillator, we implemented adjoint reflecting boundary conditions at the borders of the domain Gardiner (1985). In contrast, for the unbounded systems, since there is no natural border, we substitute the centred finite difference scheme by a forward (or backward) finite difference scheme over a bounded domain. Using adjoint reflecting boundary conditions for these systems yielded numerically very similar results.

By diagonalizing the resulting matrix, we obtain the eigenvalues and the associated eigenfunctions of $\mathcal {L}^\dagger$. We remark we are not interested in the complete spectrum of $\mathcal {L}^\dagger$ but on the small part of it (see Fig. 6). As we review at Sect. 3, we just need to consider the eigenvalue associated with the slowest decaying complex eigenfunction $Q^*_{\lambda _1}(\mathbf {x})$ to obtain the functions $u(\mathbf {x})$ and $\psi (\mathbf {x})$.

### A.2 Computing the MRT period $\overline{T}$

To compute the MRT period $\overline{T}$, we build the Kolmogorov forward operator $\mathcal {L}$ in Eq. (9) following the same numerical procedure as in A.1 underlying the construction of $\mathcal {L}^\dagger$. We obtain $P_0$ as the eigenfunction of $\mathcal {L}$ with null eigenvalue. Then, as we explained in Sect. 4, we compute the integral in Eq. (26) to obtain $\overline{T}$. More precisely, if we denote the phaseless point as $({\bar{x}}, {\bar{y}})$, we determine $\overline{T}$ by integrating the *y* component of the stationary probability current $J_{0,y}$ along the line joining ${\bar{x}}$ and $x_+$

37 $$\begin{aligned} \frac{1}{\overline{T}} = \int _{{\bar{x}}}^{x_+} J_{0,y}(x,{\bar{y}}) \mathrm{d}x. \end{aligned}$$

Of course, other choices may be possible (e.g. integrating the *x* component along the *y* axis).

### A.3 Computation of the phase offset $\varDelta \psi$

In order to obtain the phase difference $\varDelta \psi$, we interpret the system

38 $$\begin{aligned} \begin{aligned} \mathcal {L}^\dagger [\varDelta \psi (\mathbf {x})]&= 2\sum _{i,j} \mathcal {G}_{ij} \partial _i \ln (u(\mathbf {x})) \partial _j \psi (\mathbf {x}) + \frac{2 \pi }{\overline{T}} - \omega ,\\&= \varOmega (\mathbf {x}) + \varDelta \omega \end{aligned} \end{aligned}$$

as a linear system of the form $Ax = B$. Since we have already build the $\mathcal {L}^\dagger$ operator, and we already know $\omega$ and $\overline{T}$, we just need to build the terms $\varOmega (\mathbf {x})$ and $\varDelta \omega$. The computation of the derivatives of $\ln (u(\mathbf {x}))$ and $\psi (\mathbf {x})$ in $\varOmega (\mathbf {x})$ is done using a finite difference scheme.

However, solving above Eq. (38) requires to deal with two sources of numerical instability. On the one hand, the derivatives of $\ln (u(\mathbf {x}))$ and $\psi (\mathbf {x})$ diverge at the phaseless set. For this reason, the computed numerical values of $\varOmega (\mathbf {x}) + \varDelta \omega$ near the phaseless set are not accurate. On the other hand, the $\mathcal {L}^\dagger$ operator is represented by a sparse matrix, thus making the computation of its inverse numerically unstable. To overcome these numerical issues, we remove the values of $\varOmega (\mathbf {x}) + \varDelta \omega$ falling inside a small radius $r_{\text {min}}$ see (Table 1) around the phaseless set and solve Eq. (38) by means of a least squares iterative method to approximate the solution. This procedure finds a solution $\varDelta \psi (\mathbf {x})$ of Eq. (38) having a very small error (for the studied cases in this paper max error values were $\mathcal {O}(10^{-3})$). Then, we obtain an estimate for $\varDelta \psi (\mathbf {x})$ for the points of the grid inside $r_{\text {min}}$ by using extrapolation routines.

**Table 1:** Parameters for numerical implementation and resulting leading eigenvalues for the different stochastic oscillators

	*N*	*M*	$x_+$	$x_-$	$y_+$	$y_-$	$\mu$	$\omega$	$\lambda _\text {Floq}$
Sp. Sink	120	120	1.5	$-$ 1.5	1.5	$-$ 1.5	$-$ 0.080	0.564	$-$ 0.159
Wilson–Cowan	120	120	1.0	$-$ 0.3	0.9	$-$ 0.1	$-$ 0.435	1.049	$-$ 0.828
Van der Pol	120	120	2.5	$-$ 2.5	2.5	$-$ 2.5	$-$ 0.051	0.952	$-$ 0.758
Het-low	120	120	$\pi /2$	$-\pi /2$	$\pi /2$	$-\pi /2$	$-$ 0.044	0.383	$-$ 0.332
Het-high	120	120	$\pi /2$	$-\pi /2$	$\pi /2$	$-\pi /2$	$-$ 0.136	0.505	$-$ 0.553

For the noisy spiral sink in Eq. (27), we found

39 $$\begin{aligned} \varDelta \psi (\mathbf {x}) = \frac{\epsilon }{(\omega ^2 + \mu ^2)(x^2 + y^2)^2} \left( \gamma (x^2 - y^2) + 2\alpha xy \right) \nonumber \\ \end{aligned}$$

with $\gamma = -\beta _c \mu + \beta _D \omega$ and $\alpha = \beta _c \omega + \beta _D \mu$ to be a very good initial seed since it solves Eq. (38) with an $\mathcal {O}(\epsilon ^2)$ error. Thanks to this initial seed, it was not necessary to remove any point from the least square iterative solver (that is to say $r_{\text {min}}=0$).

**Fig. 7:**
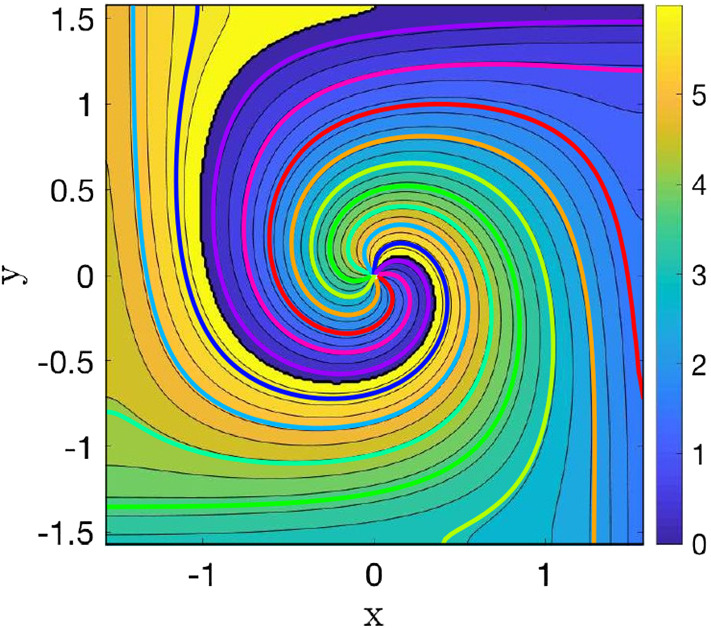
Comparison for different topologies between the MRT sections for the noisy heteroclinic oscillator with isotropic noise ($D=0.01125$). The blue-yellow colour scheme corresponds to the results published in Cao et al. (2020) (with a small hole around the origin, corresponding to an annulus). We superimposed the results of the present paper (see Fig. 4c) without a hole (hence, a topological grid). Both topologies yield numerically indistinguishable isochrons

### A.4 Topological considerations

The theoretical framework for the MRT phase requires that the domain be an annulus having inner and outer radius $[R_-, R_+]$ and implicitly assumes the existence of a phaseless point ${\bar{u}}$ (see Eq. (19)) inside the inner radius. In systems with a sufficiently high degree of symmetry, the location of the phaseless set may be clear *a priori*. For instance, in the heteroclinic oscillator, which has the symmetry of the square, the phaseless point should be at the centre of the square. However, in other systems, such as the Wilson–Cowan system, the location may not be known *a priori*. This lacuna prevents one from constructing an annular domain numerically that is guaranteed to exclude the phaseless point.

Fortunately, we have observed that in systems for which the phaseless set’s location is known, there is no practical difference in the structure of the isochrons produced by solving the MRT equation with a small central exclusion with reflecting boundary conditions, and a construction with a grid that covers the entire domain without implementing the central excluded region (see Fig. 7). This apparent robustness of the numerical procedure means that in a nonsymmetric system such as the Wilson–Cowan example, we may construct our numerical implementation *without* the inner annular exclusion. The resulting isochrons will converge and conflict in a small region on the scale of a single grid spacing. This singularity localizes the phaseless point to within the accuracy of a grid spacing.

For these reasons, despite the topological differences, we prefer to use a complete rectangular grid instead of an annulus for numerical implementation. This method leads to a simpler implementation which nevertheless still yields numerically indistinguishable results, with respect to the ones which would be obtained by explicitly adding a hole around the phaseless set. On a theoretical level, there are four separate kinds of isochrons that one may consider: the eigenvalue $\lambda$ and eigenfunction $Q=ue^{i\psi }$ for the simply connected domain, and for an annular domain; and the MRT phase $\theta$ for the annular domain and the MRT phase obtained numerically for a simply connected domain. In this paper, we investigated analytically the relationship between $\psi$ and $\theta$ on a general domain and investigated numerically the two phases on the simply connected domain. The MRT isochrons obtained using the (theoretically questionable) simply connected domain nevertheless satisfied the MRT property: the mean time to return to the isochron was independent of the starting position along the isochron.

Thus, the topological discrepancy between the annular and simply connected domain does not seem to be significant numerically, at least for the examples we considered in this paper. It is an interesting open question to ask how $\lambda$ and *Q* compare on the annular versus the simply connected domain. What impact does the size and location of the annular exclusion have on $\lambda$ and *Q*? Moreover, how do $\lambda$ and *Q* change if the hole is located at a point not overlapping the phaseless set of the simply connected domain? In cases for which we do not know *a priori* the location of the phaseless set, these questions may become highly relevant.

### A.5 Choosing the zero-th phase through the stochastic isostables

Like deterministic phase variables, the MRT phase $\varTheta$ and the stochastic asymptotic phase $\psi$ are defined up to an arbitrary additive constant. In deterministic limit cycle systems, the phase is often chosen to be zero at the maximum of some variable of interest, such as the voltage of a spiking neuron. In order to compare $\varTheta$ and $\psi$, we use a recently introduced generalization of the *isostable* coordinate adapted to stochastic systems Pérez-Cervera et al. (2021). Briefly, just as the slowest decaying *complex* eigenmode $Q^*_{\lambda _1}(\mathbf {x})$ allows one to define a stochastic phase $\psi (\mathbf {x})$, the slowest decaying purely *real* eigenmode allows one to define a stochastic amplitude since it accounts for the slowest mode describing pure *contraction* without an associated oscillation. Hence, the level curves of such a slowest decaying purely real eigenmode—which we denote as $\varSigma (\mathbf {x})$—correspond to the stochastic isostables (see Fig. 6). For this reason, and following the usual approach from deterministic systems, in this paper we used the maximum of the “stochastic limit cycle”, corresponding to the 0-level isostable $\varSigma _0\equiv \{\mathbf {x}\,|\,\varSigma (\mathbf {x})=0\}$ in the *x* direction, to set the zero phase point for both phases $\varTheta (\mathbf {x})$ and $\psi (\mathbf {x})$. In this way, we are able to provide a consistent basis for comparison throughout the paper.

### A.6 Computation of the MRT property

Once $\varDelta \psi$ is computed, we obtain the MRT phase as $\varTheta (\mathbf {x}) = \psi (\mathbf {x}) + \varDelta \psi (\mathbf {x})$. To check if the computed function $\varTheta (\mathbf {x})$ satisfies the MRT property, we do the following for each point $x_0$: first, we interpolate $\varTheta (\mathbf {x})$ in the whole domain $\mathcal {D}$ in Eq. (36) by using a 2D spline method. Then, we integrate the system of interest for a time large enough to assure that the first return occurs with overwhelming probability. In practice, we integrate for a time $3\overline{T}$ by means of a Euler–Heun scheme with a time step $\mathcal {O}(10^{-3})$. By using the interpolated grid, we can obtain a description of the trajectory $\mathbf {X}(t)$ in terms of the phase $\theta (t) = \varTheta (\mathbf {X}(t))$. Hence, we can check at which time we first cross $\varTheta (x^*) = \varTheta (x_0) + 2 \pi$. We repeat the procedure averaging the return time over $10^5$ realisations. To check the MRT property for the stochastic asymptotic phase $\psi (\mathbf {x})$, we repeat the previous procedure just substituting $\varTheta (\mathbf {x})$ by $\psi (\mathbf {x})$. The computed standard error of the MRT results in panel (e) of Figs. 1, 2, 3, 4 and 5 was found to be less than 0.1.

## B Derivation of $\frac{\mathrm{d}}{\mathrm{d}t} \mathbb {E}[\psi (\mathbf {x})]$ (Eq. 20) and $\mathcal {L}^\dagger [\psi (t)]$ (Eq. 23)

In this section, we give details of the derivation of Eqs. (20) and (23). We thank the anonymous reviewer who provided the following elegant derivation.

For the aim of a compact notation, we remove the $\mathbf {x}$ dependence for the functions, adopt Einstein’s summation convention (implicit summation over repeated indices) and derive results for $Q^*_{\lambda _1}$. First,

40 $$\begin{aligned} \begin{aligned} \mathcal {L}^\dagger [Q^*_{\lambda _1}]&= \mathcal {L}^\dagger [ue^{i \psi }], \\&= u \mathcal {L}^\dagger [e^{ i \psi }] + e^{i \psi }\mathcal {L}^\dagger [u] + 2\mathcal {G}_{jk}(\partial _ju)(\partial _k e^{ i \psi }), \\&= u \mathcal {L}^\dagger [e^{i \psi }] + e^{i \psi } \left( \mathcal {L}^\dagger [u] + 2i \mathcal {G}_{jk}(\partial _ju)(\partial _k \psi )\right) \end{aligned} \end{aligned}$$

since

41 $$\begin{aligned} \begin{aligned} \mathcal {L}^\dagger [e^{i \psi }]&= (f_j\partial _j + \mathcal {G}_{jk}\partial _j\partial _k)e^{i \psi },\\&=ie^{i \psi }f_j\partial _j\psi + i \mathcal {G}_{jk}\partial _j(e^{ i \psi } \partial _k \psi ),\\&=e^{i \psi }\left[ i\left( f_j\partial _j\psi + \mathcal {G}_{jk}\partial ^2_{jk}\psi \right) - \mathcal {G}_{jk}(\partial _j \psi ) (\partial _k \psi )\right] \\&= e^{i \psi }\left[ i \mathcal {L}^\dagger [\psi ] - \mathcal {G}_{jk}(\partial _j \psi )(\partial _k \psi )\right] \end{aligned} \end{aligned}$$

then, substituting (41) in (40) leads to

42 $$\begin{aligned} \begin{aligned} \mathcal {L}^\dagger [Q^*_{\lambda _1}]&= e^{i \psi }\left[ \mathcal {L}^\dagger [u] - \mathcal {G}_{jk}(\partial _j \psi )(\partial _k \psi ) \right. \\&\left. \quad +\, i \left( u \mathcal {L}^\dagger [\psi ] + 2\mathcal {G}_{jk}(\partial _ju)(\partial _k \psi )\right) \right] \end{aligned} \end{aligned}$$

hence, substituting $\mathcal {L}^\dagger [Q^*_{\lambda _1}] = (\mu + i\omega )ue^{i \psi }$ in (42) and dividing by $e^{i \psi }$,

43 $$\begin{aligned} \begin{aligned} \left( \mu + i\omega \right) u&= \mathcal {L}^\dagger [u] - \mathcal {G}_{jk}(\partial _j \psi )(\partial _k \psi ) \\&\quad +\, i \left( u \mathcal {L}^\dagger [\psi ] + 2\mathcal {G}_{jk}(\partial _ju)(\partial _k \psi )\right) . \end{aligned} \end{aligned}$$

Equating imaginary parts in (43) yields

44 $$\begin{aligned} u\mathcal {L}^\dagger [\psi ] + 2 \mathcal {G}_{jk}(\partial _ju)(\partial _k \psi ) = u\omega . \end{aligned}$$

Thus, wherever $u \ge 0$ is nowhere vanishing, (43) we recover

$$\begin{aligned} \mathcal {L}^\dagger [\psi ] + 2 \mathcal {G}_{jk}(\partial _j \ln (u))(\partial _k \psi ) = u\omega , \end{aligned}$$

which results in (20), (23).

For completeness, we also state

45 $$\begin{aligned} \mathcal {L}^\dagger [u] = \mu u + \mathcal {G}_{jk}(\partial _j \psi )(\partial _k \psi ) \end{aligned}$$

whose significance remains to be explored elsewhere.

## C Oscillation frequency and mean return time period for the noisy spiral sink

In this Section, we briefly discuss the details involving the derivation of the mean return period $\overline{T}$ for the two-dimensional spiral sink in Sect. 5.1.

Following Thomas and Lindner (2019), we know we can express the stationary probability density $P_0(\mathbf {x})$ as follows:

$$\begin{aligned} P_0(\mathbf {x}) = \frac{(\epsilon 2\pi )^{-1}}{ \sqrt{\det (\varPi )}} \exp \left( -\frac{ \varPi ^{-1}_{11}x^2_1 + 2 \varPi ^{-1}_{21} x_1 x_2 + \varPi ^{-1}_{22}x^2_2}{2 \epsilon } \right) \end{aligned}$$

with

46 $$\begin{aligned} \begin{aligned} \varPi ^{-1}_{11}&= -\mu \frac{(1-\beta _D)\mu ^2 + \omega ^2 - \mu \omega \beta _c}{\omega ^2 + \mu ^2(1 - \beta ^2_c - \beta ^2_D)}, \\ \varPi ^{-1}_{22}&= -\mu \frac{(1+\beta _D)\mu ^2 + \omega ^2 + \mu \omega \beta _c}{\omega ^2 + \mu ^2(1 - \beta ^2_c - \beta ^2_D)},\\ \varPi ^{-1}_{12} = \varPi ^{-1}_{21}&= \mu \frac{\beta _c\mu ^2 - \mu \omega \beta _D}{\omega ^2 + \mu ^2(1 - \beta ^2_c - \beta ^2_D)}, \end{aligned} \end{aligned}$$

and

47 $$\begin{aligned} \begin{aligned} \varPi _{11}&= \frac{(1+\beta _D)\mu ^2 + \omega ^2 + \mu \omega \beta _c}{-\mu (\omega ^2 + \mu ^2)}, \\ \varPi _{22}&= \frac{(1-\beta _D)\mu ^2 + \omega ^2 - \mu \omega \beta _c}{-\mu (\omega ^2 + \mu ^2)},\\ \varPi _{12} = \varPi _{21}&= \frac{-\beta _c\mu ^2 + \mu \omega \beta _D}{-\mu (\omega ^2 + \mu ^2)}, \end{aligned} \end{aligned}$$

with

48 $$\begin{aligned} \det (\varPi ) = \frac{\omega ^2 + \mu ^2(1 - \beta ^2_c - \beta ^2_D)}{\mu ^2(\mu ^2 + \omega ^2)}. \end{aligned}$$

Then, the probability current $\mathbf {J}$ can be written as

$$\begin{aligned} \begin{aligned} J_x(x_1, x_2) \!&=\! \left[ \mu x_1\! -\! \omega x_2 \!-\! \epsilon \left( (1+\beta _D)\partial _{x_1} \!+\! \beta _c \partial _{x_2}\right) \right] P_0(\mathbf {x}), \\ J_y(x_1, x_2)\!&= \!\left[ \omega x_1 \!+\! \mu x_2 \!-\! \epsilon \left( (1-\beta _D)\partial _{x_2} \!+\! \beta _c \partial _{x_1}\right) \right] P_0(\mathbf {x}), \end{aligned} \end{aligned}$$

so the mean period $\overline{T}$ can be computed as

49 $$\begin{aligned} \frac{1}{{\bar{T}}} = \int ^{\infty }_0 J_x(0, x_2) \mathrm{d} x_2, \end{aligned}$$

which yields

50 $$\begin{aligned} \frac{1}{\overline{T}} = \frac{w}{2 \pi \mu \sqrt{\det (\varPi )}}. \end{aligned}$$

from which we obtain $\overline{T}$ in Eq. (32).

## D Relation to the asymptotic phase for deterministic systems

In this section, we explore the relationship between the mean return time (MRT) phase $\varTheta (\mathbf {x})$, the stochastic asymptotic phase $\psi (\mathbf {x})$, and the deterministic phase $\vartheta (\mathbf {x})$. Under certain regularity assumptions, Cao et al. (2020) §2.4 established convergence of $\varTheta (\mathbf {x})$ to $\vartheta (\mathbf {x})$ in the limit of vanishing noise. Here, we present a similar argument as in Cao et al. (2020) §2.4, to establish that if suitable regularity conditions are satisfied, then in the limit of small noise the stochastic asymptotic phase $\psi (\mathbf {x})$ likewise converges to $\vartheta (\mathbf {x})$.

We start by taking the time derivative of the deterministic phase function $\vartheta (\mathbf {x})$, defined in Eq. (5),

51 $$\begin{aligned} \frac{\mathrm{d} \vartheta (\mathbf {x})}{\mathrm{d}t} = \mathbf {f}(\mathbf {x})^{\intercal } \nabla \vartheta (\mathbf {x}) = \frac{2 \pi }{T}, \end{aligned}$$

which holds $\forall \mathbf {x}$ in the basin of attraction of the limit cycle.

Consider a family of stochastic differential equations as in Eq. (7), but with the noise scaled by a small parameter $\sqrt{\epsilon }$. That is,

52 $$\begin{aligned} \frac{\mathrm{d}\mathbf {X}}{\mathrm{d}t}=\mathbf {f}(\mathbf {X}) + \sqrt{\epsilon } \mathbf {g}(\mathbf {X})\xi (t) \end{aligned}$$

where $\xi$ is a vector with components comprising independent delta-correlated white noise. Moreover, we also consider the corresponding family of solutions of Eq. (20), that is

53 $$\begin{aligned} \begin{aligned} \mathcal {L}^\dagger _\epsilon [\psi _\epsilon (\mathbf {x})]&= \mathbf {f}(\mathbf {x})^{\intercal } \nabla \psi _\epsilon (\mathbf {x}) + \epsilon \sum _{ij} \mathcal {G}_{ij} \partial ^2_{ij} \psi _\epsilon (\mathbf {x})\\&= \omega _\epsilon - 2\sum _{i,j} \mathcal {G}_{ij} \partial _i \ln (u_\epsilon (\mathbf {x})) \partial _j \psi _\epsilon (\mathbf {x}). \end{aligned} \end{aligned}$$

We now make the regularity assumption that as $\epsilon \rightarrow 0$, $\psi _\epsilon (\mathbf {x})$ converges uniformly on compact subsets of the domain to a $C^2$ function $\psi _0(\mathbf {x})$.^3^ As for any $\epsilon$, $\psi _\epsilon (\mathbf {x})$ is defined up to an additive constant, we consider convergence in the sense that for arbitrary nonzero vectors $\mathbf{v} \in \mathbb {R}^2$,

54 $$\begin{aligned} \mathbf{v} ^{\intercal }(\nabla \psi _0(\mathbf {x}) - \nabla \psi _\epsilon (\mathbf {x})) \rightarrow 0 \text {as} \epsilon \rightarrow 0, \end{aligned}$$

for all $\mathbf {x}$ in the domain. Fixing $\mathbf {x}$ and setting $\mathbf{v} = \mathbf {f}(\mathbf {x})$, we see for each $\mathbf {x}$

55 $$\begin{aligned} \mathbf {f}^{\intercal }\nabla \psi _0(\mathbf {x}) - \mathbf {f}^{\intercal }\nabla \psi _\epsilon (\mathbf {x}) = \mathbf {f}^{\intercal }\nabla \psi _0(\mathbf {x}) - \left( \omega _\epsilon + \mathcal {O}(\epsilon )\right) \rightarrow 0, \end{aligned}$$

as $\epsilon \rightarrow 0$; here, we have used Eq. (53). Consequently, if $\psi _\epsilon (\mathbf {x})$ converges to a well-behaved function $\psi _0(\mathbf {x})$ in this way, it must satisfy

56 $$\begin{aligned} \mathcal {L}^\dagger _0[\psi _0(\mathbf {x})] = \mathbf {f}(\mathbf {x})^{\intercal } \nabla \psi _0(\mathbf {x}) = \omega _0. \end{aligned}$$

Comparing Eqs. (51) and (56), evidently if the deterministic system has a stable limit cycle, then the function $\psi _0(\mathbf {x})$ must correspond with the deterministic phase $\vartheta (\mathbf {x})$ through the linear relation

57 $$\begin{aligned} \psi _0(\mathbf {x}) = \vartheta (\mathbf {x}) + \vartheta _0, \end{aligned}$$

for an arbitrary constant $\vartheta _0$.

## Footnotes

1 See Guillamon and Huguet (2009) and Pérez-Cervera et al. (2020a) for methods to obtain such parameterisation.

2 Algorithmically, we interpret $\arctan (\mathbf {x}_2/\mathbf {x}_1)$ as arctan2(x1,x2) to avoid dividing by zero.

3 Proving rigorous conditions on $\mathbf {f}$ and $\mathbf {g}$ that are either necessary or sufficient to guarantee that this assumption holds would lie beyond the scope of this paper. To our knowledge, such conditions have not yet been established in existing literature.
